# Production and Multimodal Characterization of Decellularized Extracellular Matrix from Porcine Prepubertal Tunica Albuginea as Additive to Polymeric Scaffolds for Testicular Organoid Growth

**DOI:** 10.3390/polym18020194

**Published:** 2026-01-10

**Authors:** Martina Alunni Cardinali, Iva Arato, Francesca Luzi, Marco Rallini, Cinzia Lilli, Catia Bellucci, Paola Sassi, Daniele Fioretto, Giovanni Luca, Debora Puglia, Francesca Mancuso

**Affiliations:** 1 Department of Chemistry, Biology and Biotechnology, University of Perugia, Via Elce di Sotto 8, 06123 Perugia, Italy; martina.alunnicardinali@unipg.it (M.A.C.); paola.sassi@unipg.it (P.S.); 2 Department of Medicine and Surgery, University of Perugia, Piazzale Luigi Severi, 06132 Perugia, Italy; iva.arato@unipg.it (I.A.); cinzia.lilli@unipg.it (C.L.); catia.bellucci@unipg.it (C.B.); giovanni.luca@unipg.it (G.L.); francesca.mancuso@unipg.it (F.M.); 3 Department of Science and Engineering of Matter, Environment and Urban Planning (SIMAU), Università Politecnica delle Marche, UdR INSTM, Via Brecce Bianche, 60131 Ancona, Italy; f.luzi@staff.univpm.it; 4 Civil and Environmental Engineering Department, University of Perugia, UdR INSTM, Strada di Pentima 4, 05100 Terni, Italy; marco.rallini@unipg.it; 5 Department of Physics and Geology, University of Perugia, Via Pascoli, 06123 Perugia, Italy; daniele.fioretto@unipg.it

**Keywords:** testicular organoids, chitosan, alginate, scaffold, Brillouin spectroscopy, Raman spectroscopy

## Abstract

Preservation of spermatogonial cells is of critical importance for male patients undergoing gonadotoxic therapies. Testicular organoids generated by 3D polymeric scaffolds filled with decellularized extracellular matrix (dECM) have the potential to promote stem cell growth. We propose a protocol to produce dECM from porcine prepubertal tunica albuginea for use in polymeric scaffolds. Spectroscopic analysis, molecular biology techniques, and histo-morphological assessment were used to evaluate the morphology and mechano-chemistry of the dECM at each phase of the process. The results obtained from this study demonstrate that the protocol can produce a high-purity product without causing significant alterations to protein conformation. The dECM obtained was then employed in the creation of a 3D scaffold for the cultivation of testis organoids. This was achieved by utilizing a mixture of alginate (A) and chitosan (C), which are natural polymers with a high degree of biocompatibility, that have extensive application in the field of biomedicine. Scaffold characterization demonstrated that the presence of dECM affects the scaffold’s mechanical properties by tuning structural reorganization and reducing hygroscopicity. The cell viability assay demonstrates that the A/C scaffolds are non-cytotoxic after a pre-phase of immersion in the medium.

## 1. Introduction

The preservation of fertility has become a matter of increasing importance for prepubertal male patients undergoing chemo/radiotherapy with potential gonadotoxic effects. The risk of fertility impairment associated with such therapies underscores the importance of effective preservation strategies [1,2]. In this context, the creation of testicular organoids has emerged as a potentially effective approach, as they can recreate a supportive microenvironment in which not only spermatogonial stem cells (SSCs) but also somatic cells, such as Sertoli and Leydig cells, interact synergistically to promote SSC differentiation into mature spermatozoa [3]. To achieve this objective, several proposals have been advanced, including methodologies based on the utilization of the extracellular matrix (ECM) as a biological scaffold for tissue engineering applications. ECM is a network of both structural and functional proteins assembled into unique tissue-specific architectures. It is known to play an important role in modulating cell adhesion, signaling, migration, proliferation, and three-dimensional arrangement [4]. As a result, ECM-based materials can be used in a variety of tissue engineering and regenerative medicine approaches to tissue reconstruction [5,6].

The development and differentiation of tissue into its functional form involves a myriad of coordinated and integrated pathways connecting, not only chemical, but also mechanical stimuli. Indeed, in recent decades, mechanobiology has shown how forces acting on the cell contribute to the regulation of many fundamental processes, such as cell growth, differentiation, proliferation, and motility [7]. It is therefore not surprising that the design of a scaffold suitable for *in vitro* organogenesis must consider this multifactorial complexity between the structural, chemical, and mechanical properties of the material. In this respect, attempting to recreate a niche similar to that of the native organ, i.e., the testes, could help to restore the characteristics essential for the correct differentiation and development of testicular tissue, providing a suitable environment for SSCs. Using extracellular matrix from native tissue may be effective due to its fibrous proteins, signaling molecules, and growth factors, all of which benefit organoid development. [5]. However, in order to be used as an additive, the ECM must be highly purified in terms of residual cell content. This is crucial to prevent contamination of the intended cell culture from external biological material. In this context, we propose a protocol for producing decellularized ECM (dECM) from porcine prepubertal tunica albuginea for use as an additive in polymeric scaffolds. The tunica albuginea is a dense, bluish-gray membrane with fibrous septa that provide support and contractility to the testis, thanks to a series of proteins such as elastic and collagenic fibers (Collagen I, Collagen IV), GAG (hyaluronan, heparan sulfate, keratan sulfate, chondroitin sulfate and dermatansulfate), proteoglycans (biglycan and syndecan-1), lectin and fibronectin I [8]. Because it is a dense connective tissue, it has a low percentage of cells, so the decellularization process could be simpler, and given the abundance of material, it would allow a higher yield than dECM obtained from testicular parenchyma.

Spectroscopic analysis, i.e., Brillouin and Raman micro-spectroscopy (BRamS) and ATR-FTIR spectroscopy, along with more traditional molecular biology techniques, i.e., genomic DNA and total RNA analysis, and histo-morphological assessment, were performed in order to obtain a complete evaluation of the morphology and the mechano-chemistry of each step of the dECM production process, from the original tissue to the final product. BRamS is a combined technique that can simultaneously probe the mechanical properties (Brillouin light scattering) and the chemical composition (Raman scattering) of a sample in a non-destructive and non-contact modality [9,10,11]. It has been successfully used to study the mechanobiology of single cells [12,13,14], the behavior of biomimetic hydrogels [15], and the chemo-mechanics of whole tissues [16,17,18]. ATR-FTIR is a spectroscopic technique based on the detection of infrared absorption. This technique has been demonstrated to be a valuable tool in the identification of chemical components of a given sample. Moreover, it can serve as a valuable addition to the results of a Raman spectroscopy analysis, due to its distinct sensitivity to different molecular vibrations [19,20,21].

Finally, the decellularized extracellular matrix (dECM) obtained from the TA was utilized to develop an exploratory three-dimensional scaffold for the growth of testicular organoids, in combination with mixtures of different concentrations of chitosan–alginate. Alginate and chitosan, which are natural polymers recognized for their high biocompatibility, are already widely implemented for tissue-engineering applications. Their extensively characterized properties make them an ideal and controlled platform to specifically evaluate the effects induced by the incorporation of the purified dECM into the scaffold. Furthermore, chitosan shares similarities with glycosaminoglycans (GAGs), an important chemical component of the native ECM, which are particularly relevant for the formation of a foam matrix that allows the diffusion of metabolites in the culture medium [22]. Recently, Upadhyay et al. [23] proposed a novel bioink additive (obtained by combining alginate/chitosan and decellularized ECM) in the development of scalable OOAC (Organ-on-a-Chip Articular Cartilage) using a microfluidic platform. In vitro, porous chitosan–alginate matrices have been used for investigating prostate cancer cells, glioma tumors, neural tissue development, and as a cartilage tissue-phantom [24,25,26,27]. The effect of ECM addition on the chemical and mechanical properties of these mixtures was tested, as well as their biocompatibility and their durability in culture with porcine prepubertal Sertoli cells (SCs). Salem et al. [28] reviewed the recent 2D and 3D culture systems, including both synthetic and natural (chitosan or alginate-based hydrogels), that can provide a suitable platform for male fertility preservation through organ culture of testis fragments, proliferation, and differentiation of SSCs.

## 2. Materials and Methods

### 2.1. Decellularization of Pig Testicular Tissue

Testes from prepubertal pigs were obtained from Bemoccoli Farm in Arezzo, Italy. The recovered tunica albuginea (TA) was finely chopped into fragments of about 2–3 mm^3^ and subjected to 2 different decellularization protocols as previously reported with slight modifications [29]. SD protocol: Agitation for 24 h in the presence of 0.01% SDS (Sigma–Aldrich, St. Louis, MO, USA), followed by agitation with 1% Triton X-100 (Sigma–Aldrich, St. Louis, MO, USA) for 24 h. The ST protocol, based on sequential SDS and Triton X-100 treatment, was chosen because similar low-concentration SDS/Triton workflows represent a well-established and reproducible approach for soft tissues, including testicular tissue, and have been successfully applied to generate decellularized testicular ECM suitable for in vitro applications [6]. STD protocol: Agitation for 24 h in the presence of 0.3% SDS (Sigma–Aldrich, St. Louis, MO, USA), followed by agitation for 24 h with Triton X-100 1%/0.1% ammonium hydroxide (Sigma–Aldrich, St. Louis, MO, USA). The STD protocol, which was ultimately selected for the study, extends the ST approach by incorporating an additional DNase treatment step. This modification was introduced a priori to enhance the degradation and removal of residual nucleic acids released during detergent exposure, resulting in lower residual DNA and RNA levels compared with ST alone. Thereafter, the tissue was treated for 5 h with 50 mg/mL Dnase/10 mM magnesium chloride (Sigma–Aldrich, St. Louis, MO, USA). After washing in PBS (Lonza, Verviers, Belgium), the decellularized tissue, obtained by both protocols, was sterilized for 2 h with 0.1% peracetic acid/4% ethanol solution at 4 °C (Sigma–Aldrich, St. Louis, MO, USA).

### 2.2. Porcine Prepubertal SCs Isolation

Protocols were conducted in agreement with the Italian Approved Animal Welfare Authorization (A-3143-01) and the European Community Council Directive (86/609/EEC) and approved by the University of Perugia Committee for Animal Welfare. In total, 3 Danish Duroc prepubertal pigs (15 to 20 days old) underwent bilateral orchidectomy after general anesthesia with ketamine (Ketavet 100; Intervet, Milan, Italy), at a dose of 40 mg/kg, and dexmedetomidine (Dexdomitor, Orion Corporation, Espoo, Finland), at a dose of 40 g/kg, and were used as SC donors. Specifically, pure porcine prepubertal SCs were isolated, characterized, and tested for functional competence according to previously established methods [30].

### 2.3. Powder Preparation Protocol

dECM samples, obtained from both protocols, were freeze-dried (Freez dryer, Hetovac VR1-Heto, Heto Holten, Allerød, Denmark), pulverized with a ball mill (physical shredding) by testing 4 different conditions: −5 min at 10 Hz; −5 min at 20 Hz; −5 min at 25 Hz; −5 min at 30 Hz. Aliquots of the powders were then stored at −80 °C for further analysis.

### 2.4. Alginate/Chitosan Scaffold Preparation

Different alginate (A)-based (alginic acid sodium salt from brown algae, Merck, MW = 216.12 g/mol, mannuronic acid/guluronic acid ratio = 1.56, viscosity 1% in water 15–25 cps.) and chitosan (C)-based (chitosan hydrochloride, pharmaceutical grade, Kraeber & Co GmbH, molecular weight (60 kDa), degree of deacetylation (80–90%)) scaffolds were prepared by selecting two different ratios (40:60 and 60:40 w:w, respectively) and one concentration of dECM in powder (5% w:w respect to the polymer mixture) [31]. The different formulations are summarized in Table 1.

Chitosan was first dissolved in 10 mL of deionized H_2_O at the two different concentrations (4 and 6%wt/v), at room temperature (RT) for 2 h under magnetic stirring. After that, alginate (6 and 4%wt/v, respectively) was also dissolved in the same amount of water (10 mL) and stirred for another 2 h under low-speed magnetic stirring at RT. Alginate/chitosan formulations containing dECM were prepared by dispersion of the dECM (5% wt with respect to the total amount of alginate and chitosan) in deionized H_2_O for 1 h at RT and then added to the polymers’ mixture, prepared as described above. The different A/C and A/C/dECM systems were cast on a Petri dish covered with Teflon^®^ and then immersed in different CaCl_2_ water solutions (0.5, 1, and 2%wt/v) for 15 min to obtain 3D crosslinked formulations. These polymeric scaffolds were then stored at −30 °C for 18 h and freeze-dried for 24 h.

The efficacy of decellularization was assessed by residual nuclear material quantification on hematoxylin and eosin (H&E) stained slides and by immunofluorescence analysis. Nuclei were counted in 10 random fields per slide, and results were expressed as the number of nuclei per 0.1 mm^2^. Briefly, dECM samples, produced by the two different protocols, were included in paraffin 4% paraformaldehyde (VWR, Leuven, Belgium) for 24 h at room temperature (RT). They were alcohol-dehydrated, immersed in xylene, and embedded in paraffin. Subsequently, 3–4 μm sections, obtained with a microtome (Reichert, Austria), were stained with hematoxylin and eosin (Sigma-Aldrich, St. Louis, MO, USA) and analyzed by light microscopy with a BX 41 microscope (Olympus, Tokyo, Japan) equipped with a light microscope camera (DP25, Olympus, Tokyo, Japan); images were processed with Cell F imaging software (2015 version) (Olympus, Tokyo, Japan). For the immunofluorescence analysis, 4 μm sections were obtained from the paraffin blocks obtained for histological evaluation and deparaffinized in xylene. The nuclei were counterstained with 4,6-diamino-2-phenyl (DAPI, Sigma–Aldrich, St. Louis, MO, USA) and the slides were mounted with 1,4-diazabicyclo[2.2.2]octane (DABCO, Sigma–Aldrich, St. Louis, MO, USA), before being examined with a BX 41 microscope (Olympus, Tokyo, Japan) equipped with a fluorescence photo camera (F-viewer, Olympus, Tokyo, Japan); images were processed with Cell F imaging software (Olympus, Tokyo, Japan). Genomic DNA and total RNA were isolated with Tri-reagent (Sigma-Aldrich Co., St. Louis, MO, USA) following the manufacturer’s instructions and quantified by reading the optical density at 260 nm on an Eppendorf biophotometer (Eppendorf, Enfield, CT, USA).

Prior to cell culture and MTT assays, all scaffolds were sterilized by ultraviolet (UV) irradiation under sterile conditions. Scaffolds were placed in a laminar flow hood and exposed to UV light for 30 min on each side.

Following UV treatment, the structural, biomechanical, and molecular integrity of the scaffolds was evaluated using Brillouin spectroscopy and Raman spectroscopy. These analyses confirmed that the sterilization procedure did not cause significant alterations to the dECM structure or composition. After sterilization, scaffolds were pre-wetted in complete culture medium for 24 h at 37 °C in order to equilibrate the material and remove any potential residual by-products.

### 2.5. Cell Viability Assessment

To assess the absence of any toxic substance accumulated during the scaffold preparation and decellularization stages, a 3-(4, 5-dimethylthiazol-2-yl)-2,5-diphenyl tetrazolium bromide (MTT) test was performed. For this purpose, SCs were seeded at a concentration of 4 × 10^4^ cells/well, on 96-well cell culture plates (Falcon, Glendale, AZ, USA) in 100 µL of HAMF12 (Euroclone, Milan, Italy) supplemented with 0.166 nM retinoic acid (Sigma-Aldrich Co., St. Louis, MO, USA) and 5 mL/500 mlL of Insulin-Transferrin-Selenium (ITS) + Premix (Cat. No. 354352; Corning, MA, USA), in a humidified atmosphere with 5% carbon dioxide at 37 °C. Scaffolds were pre-wetted in HAMF12 (Euroclone, Milan, Italy) for 24 h in a humidified atmosphere with 5% carbon dioxide at 37 °C and thereafter placed on 24-well cell culture plates with the same culture medium of SCs, with one change at 72 h, and stopped at 5 days. The harvested media at 72 h (T1) and 5 d (T2) were assessed for cytotoxicity, transferring 100 μL per well of SCs and incubating for an additional 48 h. Then, viability was investigated using the MTT test as previously described [32]. SCs cultured without conditioned medium served as the negative controls for cytotoxicity.

### 2.6. Spectroscopic Characterization and Data Analysis

The Brillouin and Raman micro-spectroscopy setup involves a unified configuration where the light of a 532 nm single-mode solid-state laser is focused on the sample’s surface through a microscope objective lens. The backscattered light is divided by an edge filter according to both its frequency and direction. Specifically, the Horiba iHR320 Triax Raman monochromator (HORIBA, Kyoto, Japan) receives the Stokes component (>30 cm^−1^), while the quasi-elastic and anti-Stokes components (<30 cm^−1^) are sent to the high-contrast multi-pass tandem Fabry–Perot interferometer (TFP-2 HC, JRS Scientific Instruments, Mettmenstetten, Switzerland). Both Raman and Brillouin analyses can be carried out simultaneously [33]. To prevent local photodamage, the laser power on the sample was set below 5 mW. Mapping measurements were performed using a step of 3 microns in both the *x* and *y* axes. A microscope objective 20× (NA = 0.42) was selected for both punctual and mapping analysis. Brillouin data were acquired for approximately 50 s, while Raman spectra were collected for about 60 s using a 600 lines/mm grating and a 100 μm slit aperture.

Brillouin peaks (P_BLS_) arise from the interaction between electromagnetic radiation and longitudinal acoustic modes traveling in the material, due to molecular thermal motion [9,16]. In back-scattering geometry, the frequency shift in the Brillouin peak (ν_BLS_) is related to the longitudinal elastic modulus (M_L_) of the material, by the speed of the longitudinal acoustic wave (v) via the following equation: M_L_= ρv^2^ = (ρ/4*n*^2^) (λν_BLS_)^2^, where ρ and *n* are the density of the material, λ is the wavelength of the incident light in vacuum, thus an higher frequency shift is usually associated with a stiffer material. The average Brillouin frequency shifts (ν_BLS_) were determined by computing the weighted average of the integrated signal intensity. Further details are available in ref [18].

Raman peaks arise from the interaction between incident radiation and vibrational modes of nuclei constituting the molecule. Consequently, the frequency shift in these peaks is specific for the chemical species, while the peak intensities vary proportionally with their concentration in the scattering volume [34]. Raman peak intensities (I_RAMAN_) and frequency shifts (ν_RAMAN_) are determined by integrating the signal’s area and calculating its weighted average [18].

Attenuated Total Reflection Fourier Transform Infrared (ATR-FTIR) spectroscopy is a label-free technique with a wide range of applications in the fields of biology and pharmacy. Like Raman spectroscopy, this technique is employed to monitor the chemical composition of the sample; however, it exhibits enhanced sensitivity to distinct functional groups [35,36]. Consequently, it functions as a complementary investigative tool, providing supplementary insights into the analysis. Infrared spectra were acquired using a Bruker Optics Fourier transform Alpha spectrometer and its ATR (attenuated total reflection) module equipped with a diamond crystal (Bruker Optics GmbH & Co. KG, Ettlingen, Germany). Spectra were collected in the 5000–300 cm^−1^ spectral range with 256 scans and a resolution of 2 cm^−1^.

### 2.7. SEM Analysis

The morphological characteristics of the different materials were investigated using a Field Emission Scanning Electron Microscope (FESEM, Supra 25-Zeiss, Oberkochen, Germany), gold coating the powders with an ion sputter coater, and observing the samples with the gun operating at 5 kV.

## 3. Results

### 3.1. Chemo-Mechanical Characterization of Native ECM in the Porcine Pre-Pubertal Tunica Albuginea in Hydrated and Dried Conditions

**Figure 1:**
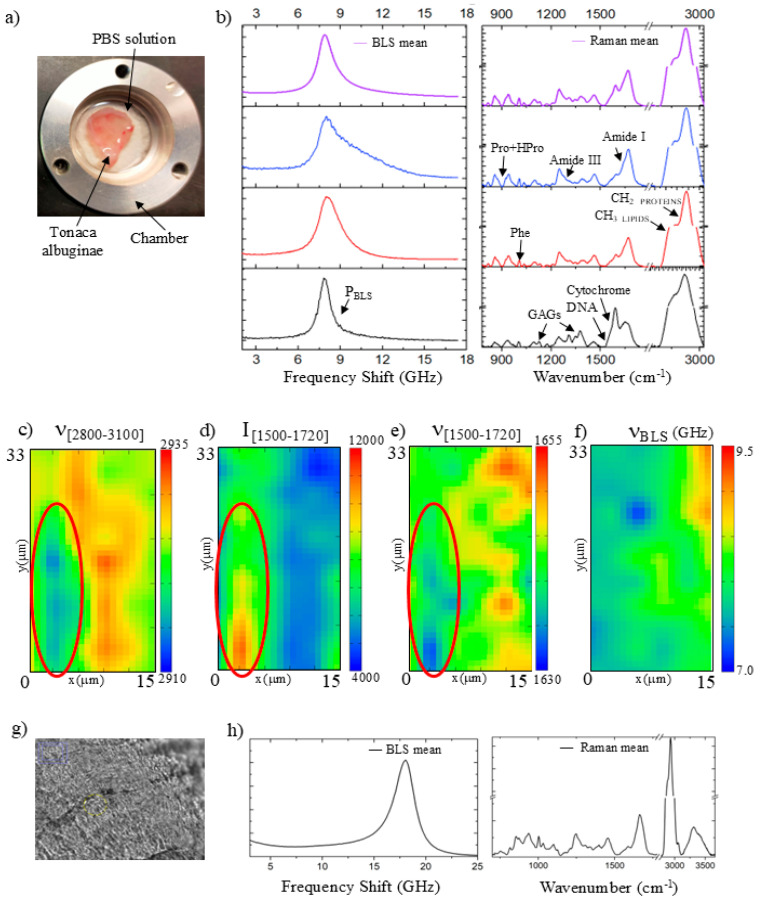
(**a**) Native porcine pre-pubertal tunica albuginea in PBS; (**b**) Brillouin (on the left) and Raman (on the right) typical spectra collected in different regions of the native tissue with peak assignations; (**c**) frequency shift contour map of CH_2_-CH_3_ region between 2800 and 3100 cm^−1^; (**d**) intensity and (**e**) frequency shift contour maps of the Amide I region between 1500 and 1720 cm^−1^; and (**f**) frequency shift contour map of the Brillouin peak. Red ellipses encircle the cellular component of the native tissue; (**g**) optical microphotograph of tunica albuginea surface without PBS (20×, NA = 0.42); and (**h**) Brillouin (on the left) and Raman (on the right) mean spectra collected on different regions of the native tissue in dried condition.

Figure 1a shows a photograph of the prepubertal porcine tunica albuginea, stored in a PBS chamber for Brillouin and Raman micro-spectroscopy analyses in hydrated conditions. As previously mentioned, this tissue provides mechanical support to the testicular parenchyma. Therefore, maintaining the native chemical and mechanical properties of the extracted extracellular matrix within the biopolymer scaffold represents a promising strategy for the cultivation of testicular organoids. Figure 1b shows selected Brillouin (left) and Raman (right) spectra from different regions probed on the surface of the sample, along with the mean spectrum (violet), which well describes the complex structure of the native tissue under hydrated conditions. Indeed, typical signatures from both the fibrous and cellular components of the TA can be recognized. The Raman spectrum shown in blue indicates a predominance of collagen due to the presence of signals from peptide bonds (Amide I and III), proline, and hydroxyproline amino acids [37,38]. Consequently, this tissue region shows a stiffness likely related to the fibers’ content, as evidenced by the Brillouin peak (P_BLS_) that has a strong shoulder shifted at high frequency. Conversely, the Raman spectrum displayed in black is indicative of the cellular component present within the tissue, which can be identified by the presence of signals attributable to DNA and cytochromes [38,39,40]. Furthermore, we also observe additional peaks that are likely associated with glycosaminoglycan (GAG) constituents of the extracellular matrix encasing these cells [41]. These areas of the tissue exhibit Brillouin spectra with a shift towards lower frequency (like that of PBS), indicating a softer material. This characteristic distinction between the features of the cells and those of the collagen fibers agrees with the results obtained for other types of tissue [18]. Finally, a significant number of spectra were found to have intermediate characteristics between these two different phenotypes (red spectra).

Figure 1c–f presents contour maps obtained from a tissue detail, showing the presence of a cell in the lower part of the map (encircled by red ellipses). The Raman spectra obtained from the cellular components reveal marked differences from the surrounding tissue. The lipid composition of the cellular membrane is signaled by a red-shift in the CH_2_-CH_3_ profile, as evidenced by the results presented in Figure 1c. Moreover, the presence of both nucleus and organelles is indicated by the increase of Raman intensity between 1500 and 1720 cm^−1^, as depicted in Figure 1d, and a simultaneous red-shift in the profile, due to the increase in the cytochrome contribution, as shown in Figure 1e. Consistent with punctual measurements, the cell is indicated by a shift to a lower value of the Brillouin frequency peak from 9.5 to about 8.5 GHz.

Finally, we conducted random measurements on the surface of the same tissue after it dried at room temperature for 24 h. Figure 1g shows an optical photograph of the tissue surface. In this case, the mean Brillouin spectrum in Figure 1h reveals an almost homogeneous spectrum with a peak at around 17.9 GHz (P_BLS_). Moreover, the corresponding Raman spectrum is very similar to the one obtained in dried human cartilage, suggesting that in this anhydrous condition, the contribution of collagen to the overall mechanical properties of the tissue is dominant [15].

### 3.2. Chemo-Mechanical Characterization, Histology, and DNA/RNA Content Evaluation of the dECM Produced via ST and STD Protocols

TA was utilized to extract dECM according to two distinct protocols, designated as ST and STD, which are described in detail in Section 2. The resulting dECM flakes were subsequently characterized using Brillouin and Raman micro-spectroscopy, histological analysis, and genomic DNA and total RNA assays to both exclude the presence of residual cells and evaluate the preservation of dECM mechano-chemical properties.

**Figure 2:**
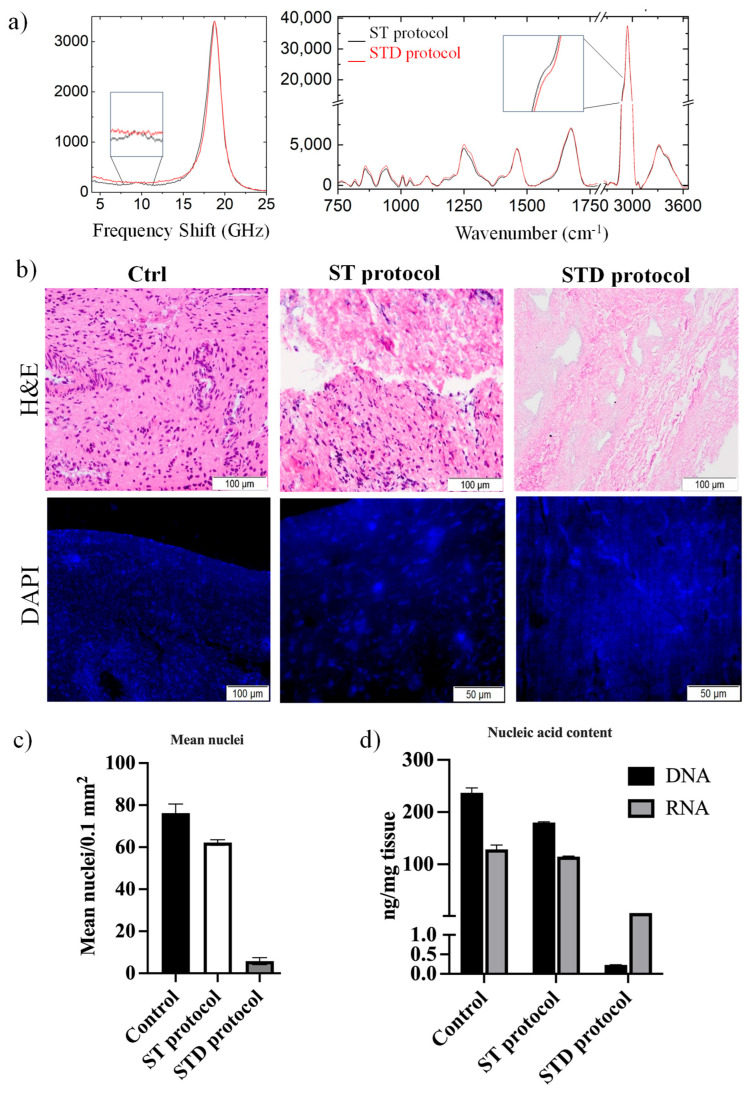
(**a**) Brillouin and Raman spectra collected on dECM flakes using the control, ST (black spectrum), and STD (red spectrum) protocols, respectively; (**b)** (upper panel) histological evaluation with H&E staining and (lower panel) immunofluorescence analysis with DAPI staining; (**c**) immunofluorescence analysis with DAPI staining in control, ST, and STD protocols; and (**d**) quantification of nucleic acid content in control, ST, and STD protocols. Genomic DNA and total RNA were extracted using Tri-reagent and quantified by UV spectrophotometry (absorbance at 260 nm). DNA and RNA values represent mean ± SD from inter-batch measurements performed on independent decellularization batches derived from different tissue pools.

Figure 2a shows the average Brillouin and Raman spectra of several points collected on the surface of the dECM flakes, obtained using the ST (black spectrum) and STD (red spectrum) protocols. The Brillouin spectra of both flakes show a clear mechanical component coming from the ECM as the dried native tissue in Figure 1h, with a peak at around 18.4 GHz, whereas the flake obtained using the ST protocol (black) shows another small peak at around 9.5 GHz. Similarly, the Raman spectra share a similar profile, but with a slight increase in the CH_2_ shoulder at 2886 cm^−1^ attributable to a higher lipid content in the ST sample (black). It is noteworthy that a BLS peak at about 9.5 GHz and the correlated presence of the Raman signal at 2886 cm^−1^ were previously identified in both dried cartilage and bone tissues, where the tissue’s cellular component is detected [18]. This suggests that the ST protocol does not entirely deplete the cellular constituents. Conversely, the STD protocol (red) provides a dECM with higher purity.

This result was confirmed by the histological evaluation that demonstrated the drastic reduction in cells with the use of the STD protocol (Figure 2 upper b,c), as indeed confirmed by immunofluorescence analysis with DAPI (Figure 2 lower b,c), which showed the presence of minimal residual nuclei compared with native TA, taken at time 0 (5.8+/−1.6 vs. 76.1+/−4.35, respectively). For the selected STD protocol, residual DNA and RNA contents were 0.23 ± 0.003 and 6.65 ± 0.06 ng/mg tissue, corresponding to coefficients of variation of approximately 1.3% (CV DNA (STD) = 0.003/0.23 × 100 ≈ 1.30%) and 0.9% (CV RNA (STD) = 0.06/6.65 × 100 ≈ 0.90%), respectively. These low coefficients of variation indicate high reproducibility of the decellularization protocol across independent batches. They were significantly lower than the TA values (237+/−9.2 and 128.62+/−8.5 ng/mg tissue, respectively), indicating that 97.91% of the nucleic acids had been removed from the tissue (Figure 2d). Therefore, the STD protocol was indicated as the most suitable protocol to achieve excellent decellularization of TA.

**Figure 3:**
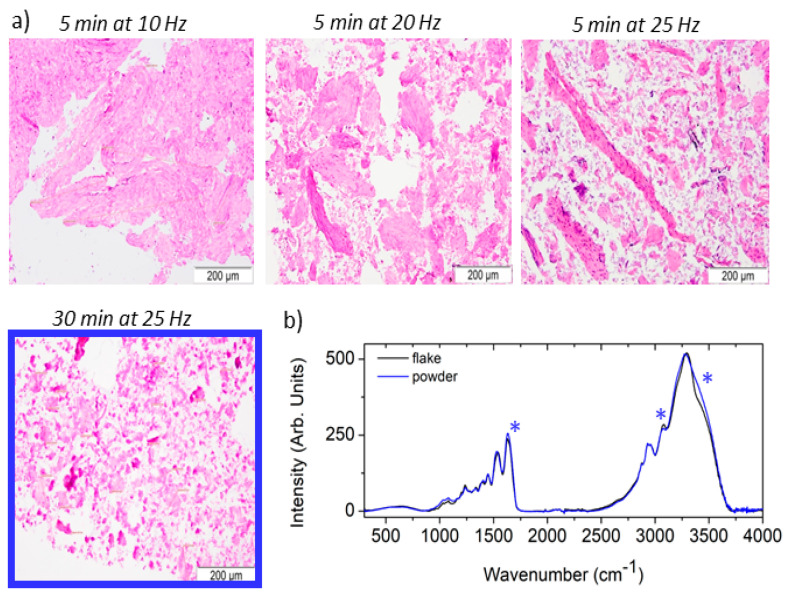
(**a**) Histological analysis using hematoxylin–eosin of SDT-dECM powders obtained by different milling protocols; (**b**) ATR-FTIR spectra on the SDT-dECM flake (black) and on the SDT-dECM powder obtained after its trituration at 25 Hz for 30 min (blue). Spectral regions with contributions from water are highlighted by asterisks.

The dECM flake obtained via the SDT protocol was further processed to be tested as an additive to produce an alginate–chitosan–based scaffold. To prepare the mixtures, dECM flakes were mechanically milled to obtain a fine powder, which was subsequently assessed histologically using hematoxylin–eosin staining, following the procedures reported in Section 2. Results showed that fragmentation with the ball mill produced a powder with a homogeneous grain size around 50+/−1.6 μm with the protocol of 5 min at 30 Hz (Figure 3a—blue square). Additionally, both the dECM flake and the different powders were analyzed using ATR-FTIR spectroscopy, which is a standard technique to fast assess the chemical composition of pharmaceuticals [20,42]. Specifically, the results reported in Figure 3b compare the mean spectrum acquired on the dECM flake (black) with that acquired on the powder triturated at 25 Hz for 30 min (blue). The ATR-FTIR spectrum of the flake confirms that the SDT-dECM matrix is primarily composed of proteins, particularly collagen type I. Furthermore, comparing the powder (blue) reveals an increase in the intensity of the OH stretching vibration at around 3500 cm^−1^ and the HOH bending vibration at around 1640 cm^−1^ (asterisks), suggesting that the powder is slightly more hygroscopic than the flake, probably due to its larger surface area exposed to ambient humidity. However, the analysis of the fingerprint region does not show any variation consistent with a change in protein structure, suggesting that the mechanical fragmentation preserves the typical fibrillar structure of collagen [43]. All the powders were found to have common spectral features, indicating that although the different trituration conditions affect powders’ granulometry, they do not substantially alter the dECM microstructure.

### 3.3. Production and Characterization of Alginate/Chitosan Scaffold

**Figure 4:**
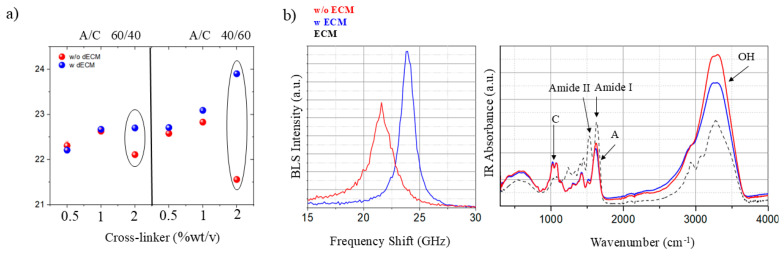
(**a**) Frequency shift in Brillouin spectra acquired on the surface of the different alginate-chitosan scaffolds with (blue dots) and without (red dots) the addition of the dECM, and (**b**) mean Brillouin and ATR-FTIR spectra collected on the surface of A/C 40/60 2%wt/v scaffold with (blue) and without (red) dECM. Ellipses denote concentrations that display divergent behavioural responses in the presence versus absence of dECM. ATR-FTIR spectra reference for the STD-dECM power was plotted in black dashed line for comparison.

Different alginate (A)- and chitosan (C)-based scaffolds were prepared considering the protocol reported in Section 2. Each sample was prepared with and without the addition of STD-dECM powder to test the effect of the presence of extracellular matrix on cell viability and on the mechano-chemical properties and structure of the scaffold. Figure 4a reports the frequency shift in Brillouin spectra acquired on the surface of A/C scaffolds, prepared with different fractions of both the two polymers and cross-linker, with (blue dots) and without (red dots) the addition of the dECM.

The overall pattern of ν_BLS_ with respect to the cross-linker concentration exhibits a comparable tendency in both A/C 40/60 (red dots on the left) and A/C 60/40 (red dots on the right) combinations, indicating an increasing stiffening of the material with increasing concentration of cross-linker. An intriguing behavior is observed in the mixtures containing higher levels of cross-linker (2%wt/v) but lacking ECM (red dots), evidenced by the black circles, which show an overall softening of the structure. This seemingly counterintuitive result may be explained by the possibility that cross-linking does not uniformly affect the entire polymer network. Localized interactions could induce structural rearrangements, leading to variations in the exposed surface (which is the one probed by the spectroscopic analysis) and, consequently, altered material properties. Furthermore, the greater deviation observed in mixtures with higher chitosan content suggests that this polymer mainly contributes to the surface softening. Indeed, chitosan is well known for forming electrostatic interactions with anionic glycosaminoglycans, proteoglycans, and other negatively charged species [44]. As demonstrated by Nasiri et al. [45], the variations in scaffold pore size can lead to reduced mechanical properties—specifically, lower stiffness—in scaffolds with higher chitosan content. Interestingly, this phenomenon does not occur when dECM is present, highlighting its critical role in determining the scaffold’s final structure.

The addition of the dECM generally results in an increase in the value of the Brillouin frequency shift (ν_BLS_), as indicated by the blue dots (Figure 4a). This increase is found to have a clear dependency on both the cross-link concentration and the relative fraction of the two polymers. Specifically, the deviation is more pronounced with higher concentrations of cross-link, and again with higher concentrations of chitosan when compared to alginate (A/C 40/60 2 mixture). To better understand the role of dECM in the polymer organization, Brillouin and ATR-FTIR spectra of the A/C 40/60 2 sample were compared in Figure 4b, for both the mixture with dECM (blue) and without (red). The ATR-FTIR spectra show characteristic intense peaks assigned to both the alginate (A) and chitosan (C) polymers [46]. It is worth noting that the spectral profiles in the fingerprint region are very similar, and the strong peaks of dECM (black dashed spectrum) in the Amide I and Amide II regions are not directly identifiable in the blue spectrum due to its low concentration. However, the observed disparities in water spectral features (i.e., the OH stretching vibration at about 3500 cm^−1^) imply a reduction in hydration within the scaffold after the integration of the dECM, which may correspond with an upward shift in Brillouin spectrum frequency. One possible explanation for the loss of hydration is that the addition of fibrous material limits polymer rearrangement caused by the crosslinker addition. This may have prevented the chitosan, the more hygroscopic component of the polymer, from being exposed on the scaffold surface, thus inducing the observed stiffening.

**Figure 5:**
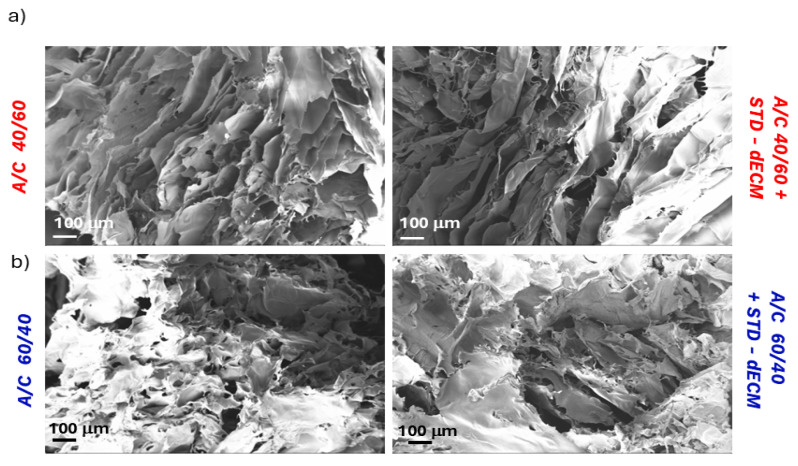
SEM images of the (**a**) A/C 40/60 2 scaffold and (**b**) A/C 60/40 2 scaffold sections before and after the addition of the STD-dECM.

The influence of dECM incorporation on the surface microstructure of the A/C 40/60 2 and A/C 60/40 2 scaffold was examined by scanning electron microscopy (SEM). Results suggest that the inclusion of dECM led to a modification of the surface. Indeed, Figure 5a,b show a rougher polymer surface in the absence of dECM, with large bundles and porosity noticeable even at the highest magnification. In contrast, a smoother and more uniform surface can be evidenced because of the inclusion of dECM. The analysis of the sections also confirmed the tuned porosities of the A/C scaffolds (A/C 40/60 is more porous than A/C 60/40) and the effect of dECM on inhibiting the formation of pores, regardless of the composition.

### 3.4. Cell Vitality Assay

**Figure 6:**
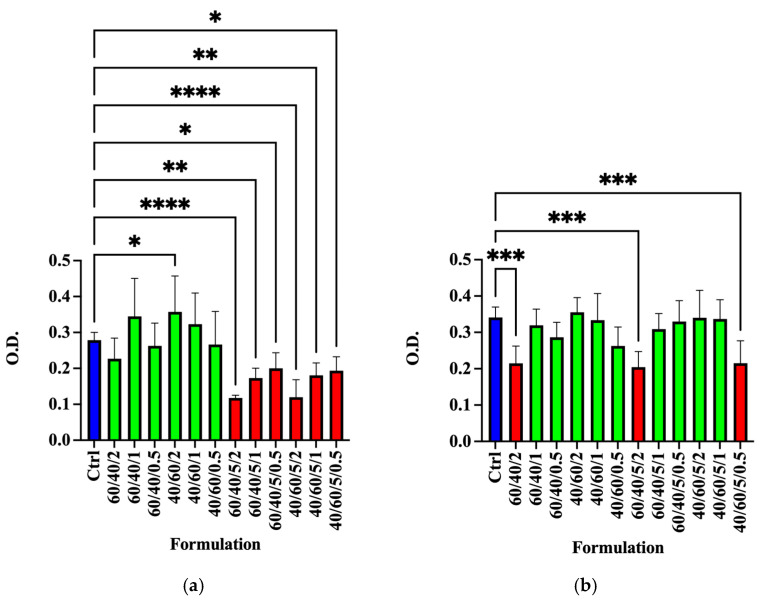
(**a**) Cell viability assessed by MTT assay using a conditioned medium from the A/C scaffold in the different formulations, with or without 5% dECM, on primary SC cultures at (**a**) T1 (72 h) and (**b**) T2 (5 d). Green indicates viable cells, whereas red indicates a statistically significant reduction in cell viability compared with the control. * *p* < 0.05, ** *p* < 0.01, *** *p* < 0.001 and **** *p* < 0.0001.

The results of the MTT test at T1 indicated that the conditioned medium of the A/C 60/40/2 scaffold and all the scaffold formulations with dECM at 5% exerted toxic effects on SCs, with respect to control samples (Figure 6a, * *p* < 0.05, ** *p* < 0.01, and **** *p* < 0.0001). This could be due to the release in harvested media in the first 72 h of cytotoxic molecules due to the dECM purification protocol or to the scaffold preparation process.

However, SC viability after five days (T2) was completely restored for all the A/C dECM formulations with respect to the control sample (Figure 6b, *** *p* < 0.001), except for the A/C 60/40/2, A/C 60/40/5/2, and A/C 40/60/5/0.5 samples, which confirmed a toxic effect of the conditioned medium. It is worth noting that both samples, A/C 60/40/2 and A/C 60/40/5/2, also exhibited the most significant variations in mechanical properties, suggesting a substantial structural rearrangement within the scaffold that could alter the diffusion of solutes in the medium. Moreover, both samples A/C 40/60/0.5 and A/C 40/60/2/0.5 showed reduced cell viability with respect to the control, indicating limited compatibility with cellular survival. Taken together, these results suggest that such formulations are not suitable for supporting cell growth, regardless of the presence or absence of dECM. This is likely due to intrinsic alterations in the scaffold induced by cross-linking and composition ratio. Indeed, these alterations seem to mediate the slow release/diffusion of cytotoxic molecules for several days.

## 4. Conclusions

In this study, we present a strategy for the extraction and purification of decellularized extracellular matrix (dECM) from the porcine prepubertal TA by using two different protocols (namely SD and SDT), to develop an additive for three-dimensional composite scaffolds designed to restore testicular spermatogenesis in vitro. The process has been characterized step by step, from the collection of TA tissue to the preparation of the additive, by combining both non-invasive spectroscopic techniques and molecular biology methods to assess dECM chemical, mechanical, and morphological properties. The analysis allows us to verify the efficacy of the SDT protocol to obtain a high-purity product, without essentially altering protein conformation.

Furthermore, a first tentative scaffold for testicular organogenesis was proposed using diverse alginate/chitosan mixtures and different concentrations of crosslinker. Spectroscopic results showed that the different relative proportions of alginate/chitosan polymers, as well as the cross-linker concentration, influence the mechanical properties of the scaffold, especially when the chitosan is the predominant component of the mixture. The incorporation of dECM has been demonstrated to result in a general increase in the stiffness of the material. A chemical evaluation revealed that these variations are likely not attributable to the formation of a new bond between the dECM and the polymer itself. Instead, they are attributed to a comprehensive reorganization of the scaffold structure, which consequently leads to a decrease in the hygroscopicity of the scaffold following the addition of dECM.

Finally, the A/C scaffolds, with and without dECM, were tested for their effect on SC viability. The results showed that, after a 72 h phase of pre-immersion in the medium, all scaffolds were non-cytotoxic compared to the control, except for the A/C 60/40/2 and A/C 40/60/5/0.5 formulations, which exhibited reduced cell viability.

## Figures and Tables

**Table 1:** Alginate/chitosan- and alginate/chitosan/dECM-based formulations.

Formulations	A (%wt/v)	C (%wt/v)	dECM (%wt/wt)
A/C 40/C60	4	6	-
A/C 60/C40	6	4	-
A/C/dECM 40/60/5	4	6	5
A/C/dECM 60/40/5	6	4	5

## Data Availability

The original contributions presented in this study are included in the article. Further inquiries can be directed to the corresponding author.
